# Development of an automatic device performing chest compression and external defibrillation: An animal-based pilot study

**DOI:** 10.1371/journal.pone.0288688

**Published:** 2023-07-26

**Authors:** Young-Il Roh, Woo Jin Jung, Hyeon Young Im, Yujin Lee, Dahye Im, Kyoung-Chul Cha, Sung Oh Hwang

**Affiliations:** Department of Emergency Medicine, Yonsei University Wonju College of Medicine, Wonju, Korea; AOU Policlinico ’Rodolico - San Marco’, ITALY

## Abstract

**Background:**

Automatic chest compression devices (ACCDs) can promote high-quality cardiopulmonary resuscitation (CPR) and are widely used worldwide. Early application of automated external defibrillators (AEDs) along with high-quality CPR is crucial for favorable outcomes in patients with cardiac arrest. Here, we developed an automated CPR (A-CPR) apparatus that combines ACCD and AED and evaluated its performance in a pilot animal-based study.

**Methods:**

Eleven pigs (n = 5, A-CPR group; n = 6, ACCD CPR and AED [conventional CPR (C-CPR)] group) were enrolled in this study. After 2 min observation without any treatment following ventricular fibrillation induction, CPR with a 30:2 compression/ventilation ratio was performed for 6 min, mimicking basic life support (BLS). A-CPR or C-CPR was applied immediately after BLS, and resuscitation including chest compression and defibrillation, was performed following a voice prompt from the A-CPR device or AED. Hemodynamic parameters, including aortic pressure, right atrial pressure, coronary perfusion pressure, carotid blood flow, and end-tidal carbon dioxide, were monitored during resuscitation. Time variables, including time to start rhythm analysis, time to charge, time to defibrillate, and time to subsequent chest compression, were also measured.

**Results:**

There were no differences in baseline characteristics, except for arterial carbon dioxide pressure (39 in A-CPR vs. 33 in C-CPR, p = 0.034), between the two groups. There were no differences in hemodynamic parameters between the groups. However, time to charge (28.9 ± 5.6 s, A-CPR group; 47.2 ± 12.4 s, C-CPR group), time to defibrillate (29.1 ± 7.2 s, A-CPR group; 50.5 ± 12.3 s, C-CPR group), and time to subsequent chest compression (32.4 ± 6.3 s, A-CPR group; 56.3 ± 10.7 s, C-CPR group) were shorter in the A-CPR group than in the C-CPR group (p = 0.015, 0.034 and 0.02 respectively).

**Conclusions:**

A-CPR can provide effective chest compressions and defibrillation, thereby shortening the time required for defibrillation.

## Introduction

Cardiopulmonary resuscitation (CPR) and defibrillation are important for survival of patients with sudden cardiac arrest [1] and an effective, high-quality CPR increases the survival rate of patients with cardiac arrest [2, 3]. Although the rate of bystander CPR is gradually increasing, the quality of CPR has been questioned. In many cases, CPR performed by ordinary individuals or even medical personnel may be insufficient [4, 5]. Automatic chest compression devices (ACCDs) can provide more uniform chest compression compared with manual chest compression [6]. As chest compressions may generate aerosols from patients with cardiac arrest, ACCDs are recommended for prolonged CPR during outbreaks of infectious disease, such as coronavirus disease 2019 pandemic [7]. Accordingly, the application of ACCDs has expanded significantly. The survival rate of patients with cardiac arrest due to ventricular fibrillation (VF)/pulseless ventricular tachycardia (pVT) is inversely proportional to the time from VF/pVT occurrence to defibrillation [8, 9]. Electrical defibrillation is the only way to terminate VF/pVT. The introduction of automated external defibrillators (AEDs) improves the survival rate of patients with cardiac arrest due to VF by enabling early defibrillation [10, 11]. Thus, ACCDs and AEDs have become essential emergency care equipment. However, carrying two equipment or treating patients with cardiac arrest may not always be feasible. In addition, because each device operates separately, it may take time to perform a series of CPR procedures. Integrating ACCD and AED can increase equipment mobility and application. Recently, we developed an automated CPR (A-CPR) apparatus that combines ACCD and AED. In this study, we evaluated the performance of the A-CPR in a pilot animal-based experiment.

## Methods

### Device description

**Fig 1:**
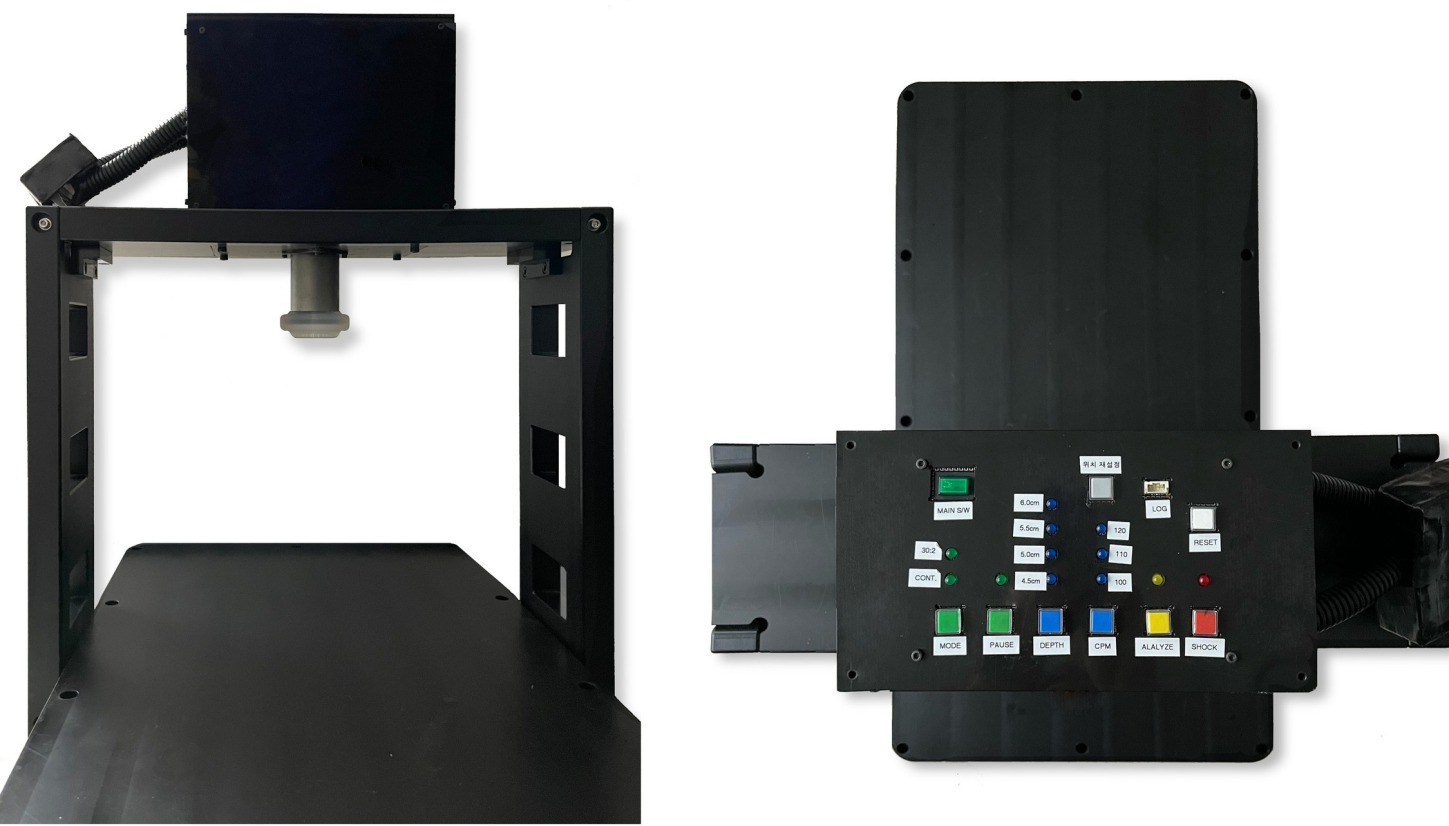
Automated cardiopulmonary resuscitation apparatus.

**Fig 2:**
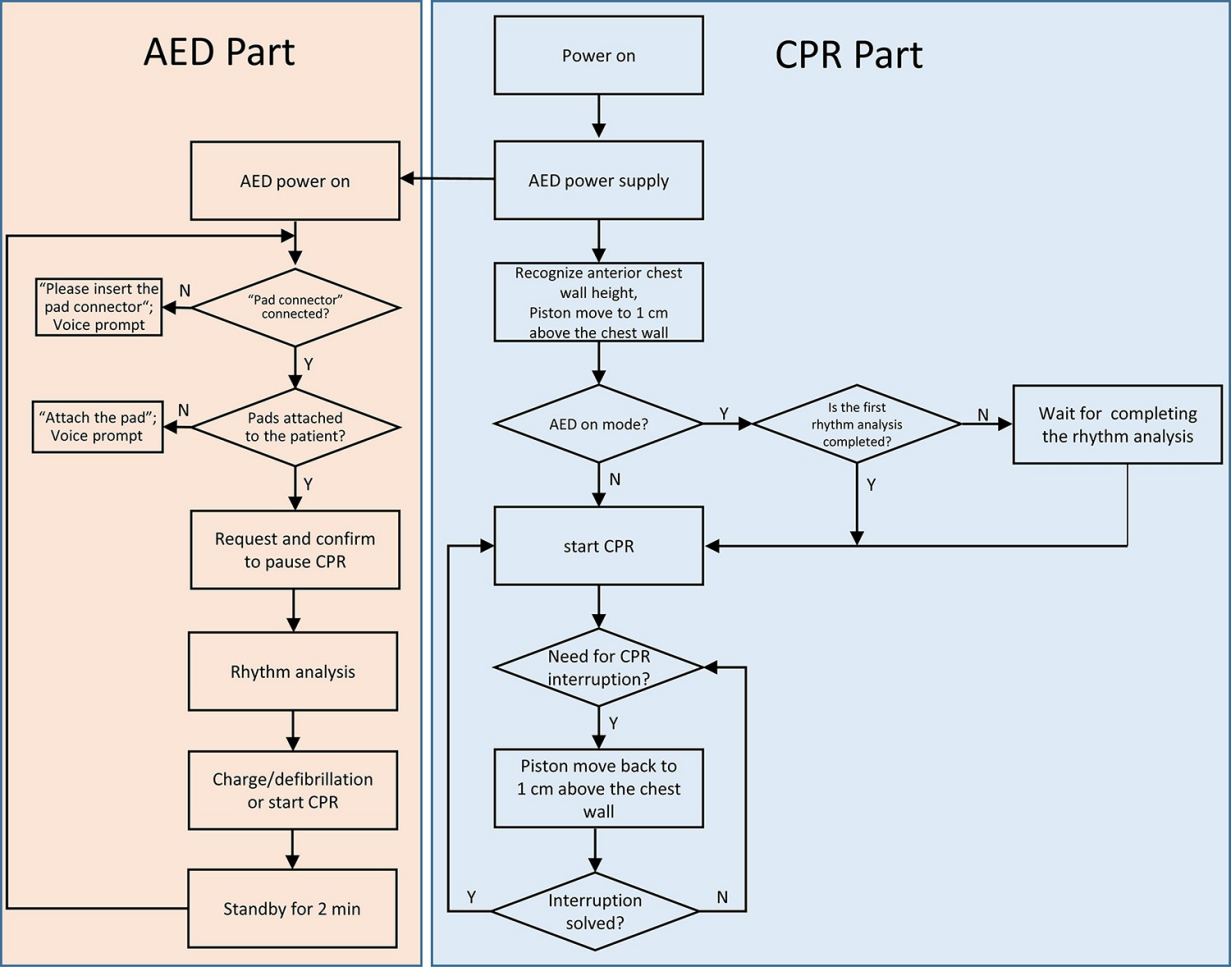
Algorithm of an automated cardiopulmonary resuscitation sequence.

A-CPR consists of a functional part and a supporting frame (**Fig 1**). The functional part is mounted on the supporting frame, which is composed of a compression-performing apparatus, an AED, and a control panel. The compression-performing apparatus is composed of a piston and an actuator and is used to compress the chest. The piston is a round bar connected to the actuator operated by a battery. The compression depth and rate of compression range from 0 to 6 cm and 0 to 120 compressions per minute, respectively, and can be adjusted. An AED is integrated with the compression-performing apparatus. The defibrillation electrodes are stored in the designated pocket. The control panel controls chest compression and automated defibrillation. The operator can control the start or stop of chest compression, compression depth, compression rate, and shock delivery. The support frame consists of a backboard and a supporting structure on which the compression-performing apparatus and AED are mounted. The sequence of chest compression and defibrillation is based on the basic life support (BLS) algorithm and is installed into the A-CPR [3, 12]. Once the A-CPR is initiated, it first analyzes the rhythm. If a shockable rhythm is detected, the A-CPR starts chest compression and recommends performing defibrillation by pressing the shock button while continuing chest compression. If a non-shockable rhythm is detected, the A-CPR immediately performs mechanical chest compressions (**Fig 2**).

### Pilot animal study

This study was designed to evaluate A-CPR performance and compare it with that of conventional CPR (C-CPR) performed using an ACCD and an AED in a swine model of cardiac arrest. This study was approved by the Institutional Animal Care and Use Committee of Yonsei University Wonju College of Medicine, Wonju, Republic of Korea (YWC-210517-1).

### Animal preparation

Twelve Yorkshire pigs (weight, 35–43 kg; six females) were used in this study. The pigs were allowed full access to water and food until the day before the experiment and were fasted from midnight. After sedating the animals with intramuscular ketamine (15 mg/kg) and xylazine (2 mg/kg) followed by inhalation of 3% isoflurane, endotracheal intubation was performed with a cuffed endotracheal tube. The animals were ventilated with oxygen and nitrous oxide via a volume-controlled ventilator (Draeger Fabius GS, Draeger Medical Inc., Telford, PA) with a tidal volume of 10 mL/kg and a ventilation rate of 18 breaths/min adjusted to maintain normal arterial oxygen saturation (94–98%) and end-tidal carbon dioxide (ETCO_2_) (35–45 mmHg). Under aseptic conditions, the right femoral artery was cannulated, and aortic blood pressure was continuously recorded using a 5-F micromanometer-tipped catheter. Right atrial pressure was recorded using a 5-F micromanometer-tipped catheter inserted through the right external jugular vein. A vascular flowmeter (Transonic Systems, Inc., Ithaca, NY, USA) was placed on the right internal carotid artery to monitor carotid blood flow (CBF). A 5-F pacing catheter was introduced through the right internal jugular vein to induce VF. Once the catheters were in place, a 1,000 unit intravenous (IV) heparin bolus was administered to prevent thrombosis, and baseline arterial blood gas analyses were performed using a blood gas analyzer (i-STAT1, Abbott Laboratories, Abbott Park, IL).

### Study protocol

The pigs were randomized into two groups using randomization envelopes containing different CPR methods (A-CPR or C-CPR group). An ACCD or A-CPR was positioned before the experiment to minimize the risk of catheter or the monitoring apparatus dislodging. AED (AED Plus^®^, Zoll, Chelmsford, Mass, USA) and pads were also applied before the start of the experiment to reduce the bias from any delay in applying AED pads.

**Fig 3:**
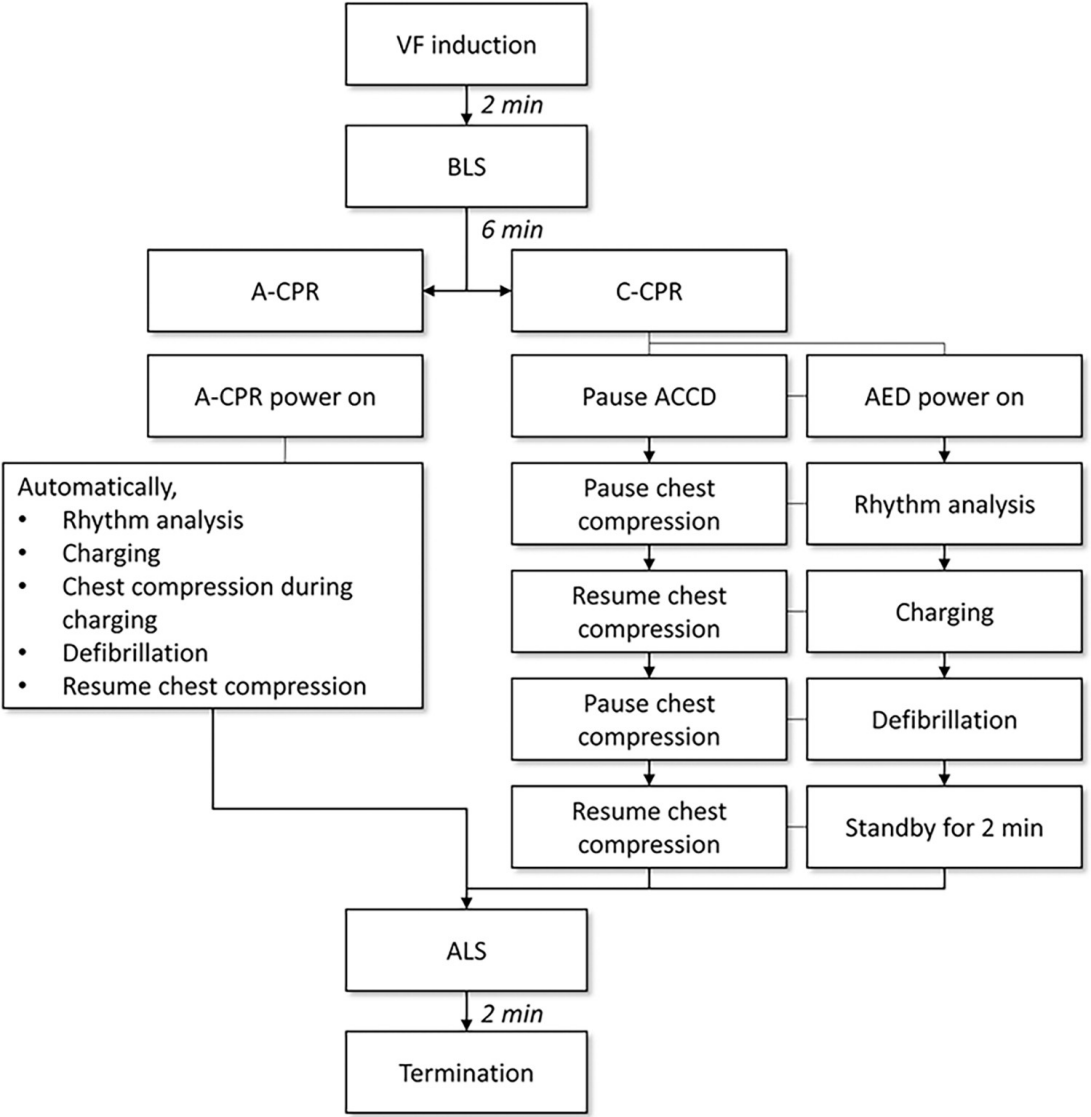
Algorithms of automated cardiopulmonary resuscitation (CPR) and conventional CPR.

After the baseline data measurement, VF was induced by delivering an alternating electrical current of 60 Hz to the endocardium, which was confirmed by the electrocardiogram (ECG) waveform and a decrease in aortic pressure. After 2 minutes of untreated VF, basic BLS was performed for 6 minutes to mimic a BLS situation in which a bystander recognizes cardiac arrest and calls for help. Mechanical chest compressions with a depth of 5 cm using an ACCD (LUCAS2, Stryker Medical, Kalamazoo, MI, USA) or A-CPR were performed at a rate of 100 chest compressions/min and a compression/ventilation ratio of 30:2 was maintained. Positive pressure ventilation with a room air at a tidal volume of approximately 300 mL was delivered using a resuscitator bag (Silicone resuscitator 87005133, Laerdal Medical, Stavanger, Norway). After 6 min of BLS, defibrillation was performed according to the instructions (voice prompt) from the AED for the C-CPR group and the A-CPR device for the A-CPR group. After defibrillation, chest compression administered continuously while single ventilation with 100% oxygen were performed every 6 s, mimicking advanced life support (ALS) (**Fig 3**).

The experiment was terminated immediately after 2 min of ALS. Once a pig achieved return of spontaneous circulation (ROSC), the animal was euthanized by IV potassium chloride injection.

### Data measurements

Data were digitized using a digital recording system (PowerLab, AD Instruments, Colorado Springs, CO, USA). Aortic and RAP, ETCO_2_, and CBF were continuously recorded. Coronary perfusion pressure (CPP) was calculated as the difference between the aortic and right atrial pressures in the end-diastolic phase. Outcome measures included chest compression fraction (CCF), rate of successful defibrillation, and ROSC. Time variables, including time to start rhythm analysis, time to charge, time to defibrillation, and time to subsequent chest compression, were measured.

### Data analysis

Continuous variables are presented as mean ± standard deviation or medians and interquartile ranges according to the normality of distribution. Student’s *t*-test or the Mann–Whitney U test was used to compare the continuous variables between the A-CPR and C-CPR groups, as appropriate. The nominal variables are reported as counts and percentages and were compared using the chi-square or Fisher exact test, as appropriate. A linear mixed-model analysis was used to compare hemodynamic parameters, including aortic systolic pressure, aortic diastolic pressure, mean RAP, CBF, CPP, and ETCO_2_ between the two groups. The statistical results are presented as group-time interaction. Results with P < 0.05 were considered statistically significant. Data were analyzed using the Statistical Package for the Social Sciences Statistics version 23.0 for Windows (IBM Corp., Chicago, IL, USA).

## Results

### Baseline characteristics

Initially, six pigs were enrolled in each group; however, one in the A-CPR group was excluded because of AED malfunction. Thus, five pigs in the A-CPR group and six pigs in the C-CPR group were included in the final analysis. There were no significant differences, except PaCO_2_, in baseline characteristics between the groups (**Table 1**).

**Table 1:** Baseline characteristics.

	A-CPR (n = 5)	C-CPR (n = 6)	P value
Weight (kg)	39 ± 1.5	39 ± 3.2	0.722
Female, n (%)	2 (40)	3 (50)	1.000
AoS (mmHg)	109 (103–121)	110 (95–121)	0.931
AoD (mmHg)	74 (70–80)	78 (59–86)	0.856
MAP (mmHg)	86 (85–101)	90 (71–97)	0.421
RAS (mmHg)	7.9 (5.3–8.5)	7.0 (5.8–9.1)	0.931
RAD (mmHg)	2 (0–3)	1 (-1–3)	0.841
CPP (mmHg)	81 (76–85)	88 (74–99)	0.269
CBF (mL/min)	191 (154–253)	263 (142–422)	0.383
ETCO_2_ (mmHg)	47 (44–52)	45 (42–48)	0.257
pH	7.487 (7.433–7.541)	7.532 (7.522–7.545)	0.114
PaCO_2_ (mmHg)	39 (35–40)	33 (31–36)	0.034
PaO_2_ (mmHg)	120 (105–161)	145 (117–167)	0.539
HCO_3_^-^ (mmol/L)	28 (27–31)	29 (26–32)	0.649
SaO_2_ (%)	99 (98–100)	99 (99–100)	0.527
Lactate (mmol/L)	2.1 (1.0–2.9)	1.8 (1.2–2.6)	0.843

Variables are presented as medians (interquartile ranges) or frequencies (percentages).

A-CPR, automatic cardiopulmonary resuscitation; C-CPR, conventional cardiopulmonary resuscitation; AoS, aortic systole; AoD, aortic diastole; RAS, right atrial systole; RAD, right atrial diastole; MAP, mean arterial pressure; CPP, coronary perfusion pressure; ETCO_2_, end-tidal carbon dioxide; CBF, carotid blood flow; ROSC, return of spontaneous circulation; PaCO_2_, arterial pressure of arterial carbon dioxide; PaO_2_, arterial pressure of arterial oxygen; HCO_3_^-^, bicarbonate; SaO2, arterial oxygen saturation

### Hemodynamic parameters during cardiopulmonary resuscitation

**Fig 4:**
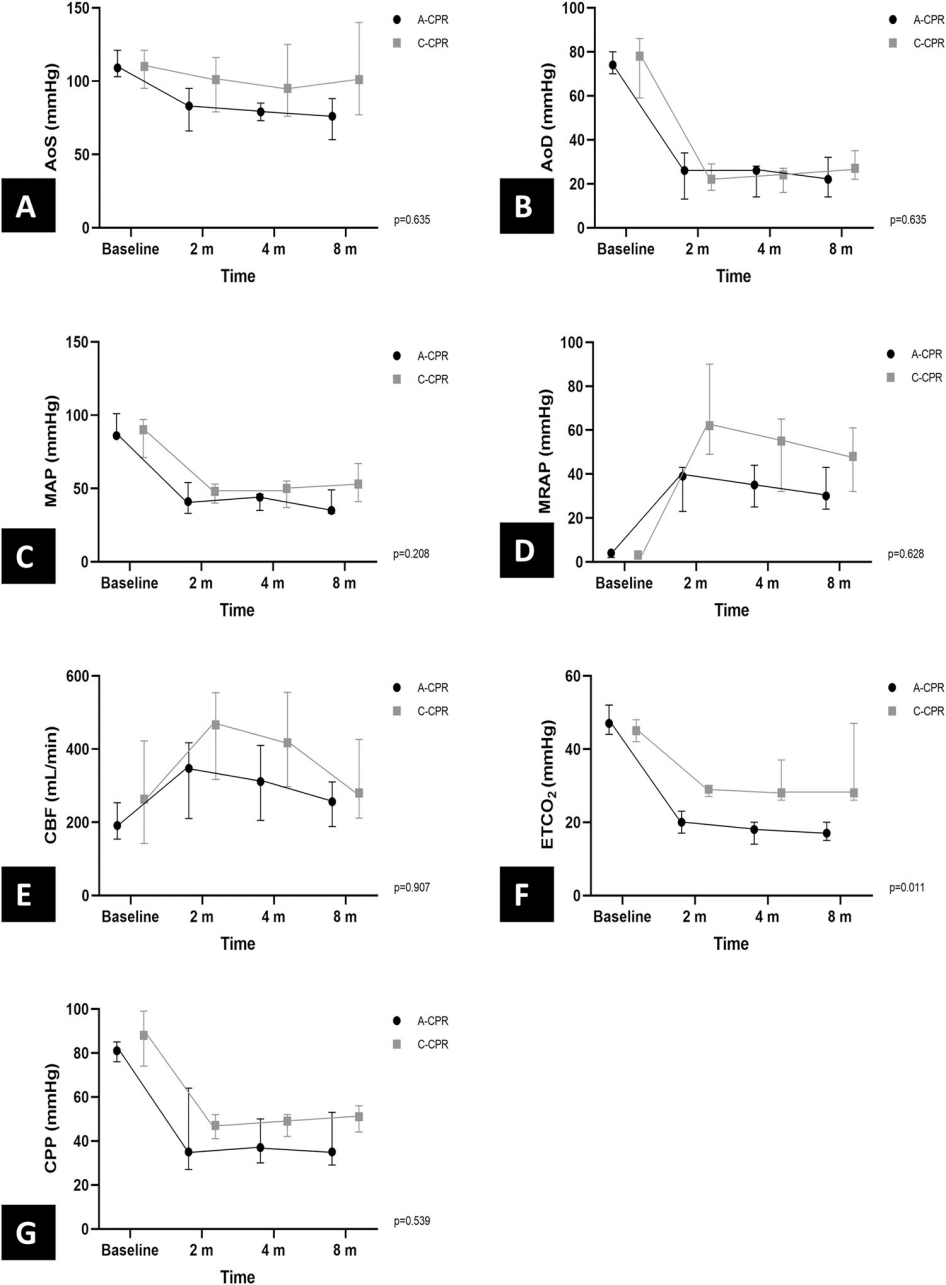
Comparison of hemodynamic parameters during cardiopulmonary resuscitation.

There were no significant differences between the groups in the group-time interaction analyses of hemodynamic variables (**Fig 4**).

### Outcomes

There was no significant difference in CCF between the groups (81.7 ± 1.1%, A-CPR group; 81.1 ± 1.4, C-CPR group; p = 0.418). There were no differences in ROSC and successful defibrillation because spontaneous circulation was restored in all animals after the first defibrillation and after 2 min of ALS.

### Comparison of time variables

Time to charge (28.9 ± 5.6 s in the A-CPR group vs. 47.2 ± 12.4 s in the C-CPR group), time to defibrillation (29.1 ± 7.2 s in the A-CPR group vs. 50.5 ± 12.3 s in the C-CPR group), and time to subsequent chest compression (32.4 ± 6.3 s in the A-CPR group vs. 56.3 ± 10.7 s in the C-CPR group) were shorter in the A-CPR group than in the C-CPR group (p = 0.015, 0.034, and 0.02, respectively) (**Table 2**).

**Table 2:** Comparison of chest compression fraction and time variables between the groups.

	A-CPR (n = 5)	C-CPR (n = 6)	P value
CCF (%)	81.7 ± 1.1	81.1 ± 1.4	0.418
Time to start rhythm analysis (s)	23.7 ± 4.9	32.5 ± 11.9	0.161
Time to charge	28.9 ± 5.6	47.2 ± 12.4	0.015
Time to defibrillation	29.1 ± 7.2	50.0 ± 12.3	0.034
Time to subsequent chest compression	32.4 ± 6.3	56.3 ± 10.7	0.002

Variables are presented as mean ± standard deviation.

A-CPR, automatic cardiopulmonary resuscitation; C-CPR, conventional cardiopulmonary resuscitation; CCF, chest compression fraction

## Discussions

The A-CPR developed in this study demonstrated that it is feasible to integrate an AED with an ACCD. Furthermore, we showed that A-CPR was more efficient to reduce the time to charge, defibrillation and subsequent chest compression in an animal model of cardiac arrest than that obtained using the two equipment individually.

In animals with VF, cessation of chest compressions for > 15 s before defibrillation compromised CPR outcomes [13]. In clinical observations, the duration of the single longest pause in chest compression is associated with unfavorable outcomes in patients with out-of-hospital cardiac arrest (OHCA) [14]. When the pulse is assessed after defibrillation, subsequent chest compression can be delayed by up to 29 s [15]. Accordingly, the CPR guidelines recommend starting chest compression immediately after defibrillation and minimizing interruptions in chest compressions during CPR [3, 12]. However, pause in chest compressions before and after electrical shock delivery in patients with cardiac arrest negatively impacts patient survival rate [16]. Chest compressions are frequently interrupted during endotracheal intubation to analyze the rhythm, charge the defibrillator, perform defibrillation, check the pulse, move the patient, initiate CPR devices, shift compressors, and take over by healthcare providers [17–20]. Using a device that integrates an ACCD with an AED may reduce interruptions in chest compressions. A-CPR is designed to resume chest compressions immediately after ECG analysis and continuous chest compressions during defibrillation so that chest compressions are not interrupted during CPR, except for the time required for the ECG analysis. In this pilot study, when A-CPR was used, the time to defibrillation was shortened by approximately 11 s and the time to subsequent chest compression was shortened by approximately 14 s compared to when an AED and an ACCD were used separately. In patients with OHCA from VF, the likelihood of ROSC increases when the pre-shock interval is < 3 s and the post-shock interval is < 6 s [16]. Time to defibrillator charge was also shortened by 20 s with A-CPR compared to that with C-CPR. Therefore, decreased interruptions in chest compression using A-CPR may have a favorable effect on the outcome of cardiac arrest.

Healthcare providers have to carry several equipment for resuscitation to the scene of cardiac arrest, including AEDs and ACCDs, and perform several resuscitation steps including intubation, ventilation, chest compressions, and defibrillation. A-CPR may increase procedural efficiency and patient outcomes by integrating the functionalities of an ACCD and an AED in a single device.

Transthoracic impedance is affected by shock waveforms, coupling devices, electrode size and position, respiration, lung volume, and respiration phase [21]. High transthoracic impedance necessitates multiple shocks and greater energy delivery for successful defibrillation [22]. Higher lung volume during inspiration may cause longer current paths, resulting in an increase in transthoracic impedance [23]. Transthoracic impedance is 9% lower at the end of expiration than at the end of inspiration [24]. Thus, delivering shock during inspiration significantly lowers the defibrillation success rate compared with delivering the shock during expiration [25]. A-CPR delivers defibrillation energy without interrupting chest compressions and delivers shock at the end of the compression phase. Lung volume is smaller during the compression phase than that during the relaxation phase. Hence, shock delivery by A-CPR may favorably influence defibrillation success.

### Limitations

As the A-CPR used in this experiment was manufactured on the premise of its use in humans, there were some limitations in its application to animals. This pilot study evaluated the in vivo operation of A-CPR and compared the temporal benefit between the use of A-CPR and the separate use of an AED and an ACCD in a small number of animals. Therefore, there were limitations in comparing resuscitation outcomes between the two groups. To prevent the catheter inserted into the experimental animal from dislodging, the experiment was conducted with A-CPR or ACCD placed on the animal in advance. Hence, these results may not be completely generalized to humans and may need further validation before this device can be used in clinical settings.

## Conclusions

An automatic CPR device developed in this study integrates the functions of mechanical chest compressions and defibrillation to achieve significant hemodynamic outcomes. A-CPR can provide continuous chest compressions during defibrillation, thereby shortening the time required for defibrillation and improving treatment outcomes.

## Supporting information

S1 Checklist The ARRIVE guidelines 2.0: Author checklist. (PDF)
